# S100A9 is a functional effector of infarct wall thinning after myocardial infarction

**DOI:** 10.1152/ajpheart.00475.2021

**Published:** 2021-12-10

**Authors:** Upendra Chalise, Mediha Becirovic-Agic, Michael J. Daseke, Shelby R. Konfrst, Jocelyn R. Rodriguez-Paar, Dan Feng, Jeffrey D. Salomon, Daniel R. Anderson, Leah M. Cook, Merry L. Lindsey

**Affiliations:** ^1^Department of Cellular and Integrative Physiology, Center for Heart and Vascular Research, University of Nebraska Medical Center, Omaha, Nebraska; ^2^Research Service, Nebraska-Western Iowa Health Care System, Omaha, Nebraska; ^3^Department of Physiology and Biophysics, University of Mississippi Medical Center, Jackson, Mississippi; ^4^Division of Pediatric Critical Care, Department of Pediatrics, University of Nebraska Medical Center, Omaha, Nebraska; ^5^Division of Cardiovascular Medicine, Department of Internal Medicine, University of Nebraska Medical Center, Omaha, Nebraska; ^6^Department of Pathology and Microbiology, University of Nebraska Medical Center, Omaha, Nebraska

**Keywords:** infarct wall thinning, inflammation, myocardial infarction, neutrophil, S100A9

## Abstract

Neutrophils infiltrate into the left ventricle (LV) early after myocardial infarction (MI) and launch a proinflammatory response. Along with neutrophil infiltration, LV wall thinning due to cardiomyocyte necrosis also peaks at *day 1* in the mouse model of MI. To understand the correlation, we examined a previously published data set that included *day 0* (*n* = 10) and MI *day* (*D*) *1* (*n* = 10) neutrophil proteome and echocardiography assessments. Out of 123 proteins, 4 proteins positively correlated with the infarct wall thinning index (1/wall thickness): histone 1.2 (*r* = 0.62, *P* = 0.004), S100A9 (*r* = 0.60, *P* = 0.005), histone 3.1 (*r* = 0.55, *P* = 0.01), and fibrinogen (*r* = 0.47, *P* = 0.04). As S100A9 was the highest ranked secreted protein, we hypothesized that S100A9 is a functional effector of infarct wall thinning. We exogenously administered S100A8/A9 at the time of MI to mice [C57BL/6J, male, 3–6 mo of age, *n* = 7 M (*D1*), and *n* = 5 M (*D3*)] and compared with saline vehicle control-treated mice [*n* = 6 M (*D1*) and *n* = 6 M (*D3*)] at MI *days 1* and *3*. At MI *day 3*, the S100A8/A9 group showed a 22% increase in the wall thinning index compared with saline (*P* = 0.02), along with higher dilation and lower ejection fraction. The decline in cardiac physiology occurred subsequent to increased neutrophil and macrophage infiltration at MI *day 1* and increased macrophage infiltration at *D3*. Our results reveal that S100A9 is a functional effector of infarct wall thinning.

**NEW & NOTEWORTHY** S100A9 is a functional marker of infarct wall thinning.

## INTRODUCTION

The response to myocardial infarction (MI) involves a robust immune response with inflammatory cell infiltration initiated within the first hours after coronary artery occlusion. Proinflammatory neutrophils and monocytes are early responders to danger-associated molecular patterns (DAMPs) released from necrotic cardiomyocytes (1–3). Leukocytes infiltrate the infarct regions and commence the inflammatory cascade by secreting cytokines, chemokines, and proteases essential for extracellular matrix (ECM) breakdown and remodeling. Excessive infiltration of proinflammatory cells after MI can have a negative impact on cardiac remodeling and repair mechanisms (4, 5). Although tempering the proinflammatory cascade will curb excessive cardiomyocyte and ECM turnover, proinflammation is an essential preceding guide for later anti-inflammatory and reparative signaling. For this reason, anti-inflammatory strategies that totally block proinflammation have often resulted in negative impacts on cardiac remodeling (6).

Cardiomyocyte necrosis and subsequent loss after MI defines the magnitude of infarct wall thinning that occurs (7, 8). In the mouse MI model, maximum myocyte loss occurs within 24 h of ischemia initiation, with wall thinning peaking at *day* (*D*) *1*. Infarct wall thinning can be measured in vivo by echocardiography. Wall thinning weakens cardiac structure and can lead to formation of left ventricle (LV) aneurysms, which can further progress to LV rupture. LV rupture, while less prevalent clinically due to the advent of reperfusion, is still a major cause of MI mortality in preclinical MI animal models and clinically with failed reperfusion or ineffective cardiac repair (9, 10). About 25% of MI cases in humans are nonreperfused due to inaccessibility or delayed presentation of MI or other medical contraindications. An additional ∼30% of MI cases undergoing percutaneous intervention fail and are categorized as no reflow (11, 12). No reflow accounts for ∼500,000 patients yearly in the United States alone (11, 12). In both of these cases, the patient is at high risk for adverse cardiac remodeling that progresses to heart failure. In mice, the nonreperfused MI model best replicates this clinical scenario.

Neutrophil numbers positively associate with LV rupture (13). Neutrophils secrete a variety of proteases including serine proteases, matrix metalloproteinases (MMPs), and neutrophil elastase to orchestrate the removal of necrotic debris. The neutrophil expresses several MMPs, particularly MMP-8 and MMP-9, which are stored in preformed granules (14). MMP-8 and MMP-9 proteolytically cleave a number of substrates including ECM, chemokines, and cytokines. Increased MMP-8 or MMP-9 in the infarct zone associates with LV rupture and heart failure progression after MI (9, 15–17).

The S100A8/A9 complex, also known as calprotectin, is a DAMP secreted by neutrophils and monocytes and regulates inflammatory responses through its role in innate immunity (18). The two subunits belong to the S100 family of proteins, which regulates various cellular functions including calcium balance, cellular migration, proliferation, and inflammation (19). S100A8 and S100A9 combined account for ∼45% of total cytosolic protein content in neutrophils. S100A9 is a calcium-binding protein that primarily exists as a heterodimer with S100A8.

The goal of this study was to identify proteins derived from the neutrophil that best correlate with infarct wall thinning and could serve as causal mediators. Our results identified four proteins, of which S100A9 was the highest extracellular component. We then tested the hypothesis that as a direct regulator, S100A9 administration after MI would increase infarct wall thinning.

## MATERIALS AND METHODS

### Identification of Protein Candidates

**Figure 1.**
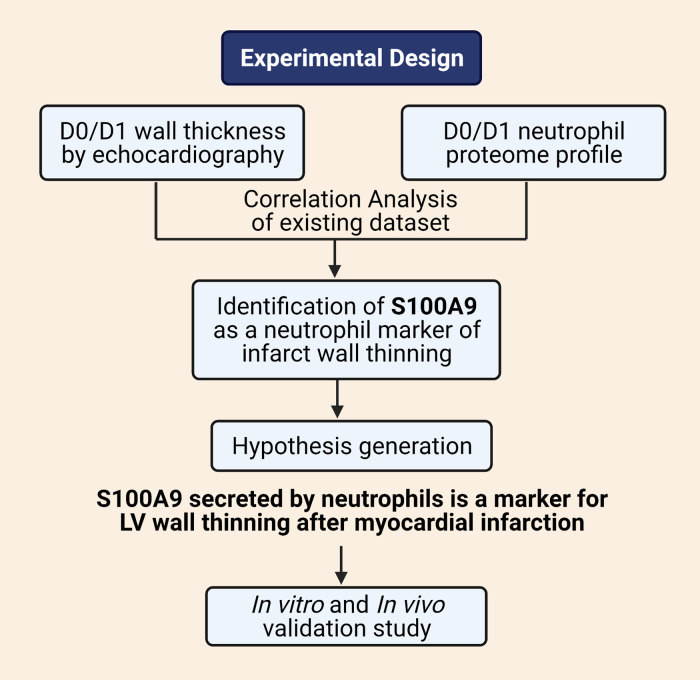
Experimental design. Created with BioRender.com and published with permission.

The experimental design workflow is detailed in Fig. 1. We retrospectively interrogated a previously published MI neutrophil proteome and echocardiography data set to identify proteins that best linked to infarct wall thinning (8). The data set included 123 neutrophil proteins, which were correlated with infarct wall thinning (anterior wall thickness in systole, AWTs) calculated as the inverse of wall thickness (1/AWTs) measured by echocardiography (8). The data set included results for 3- to 6-mo-old C57BL/6J male mice at control no MI *D0* (*n* = 10) or MI *D1* (*n* = 10). Neutrophils were isolated from the infarct region at MI *D1* and compared with no MI *D0* LV neutrophils. By Pearson correlation analysis, the correlation coefficient (*r*) and *P* value were used to determine ranking. Positive correlations between wall thinning and protein concentration were used for target selection and hypothesis generation for the prospective study.

### Coronary Artery Ligation and Echocardiography

For the prospective evaluation, all animal procedures were approved by the Institutional Animal Care and Use Committee at the University of Nebraska Medical Center and were conducted in accordance with the *Guide for the Care and Use of Laboratory Animals*, published by the National Research Council (20). We enrolled a new set of mice for validation assessment. C57BL/6J male mice (3- to 6-mo old) were used for MI surgery using the same protocol, as previously described (5, 21–25). Isoflurane (∼2%) was used for anesthesia, and buprenorphine-SR (0.5 mg/kg sc) was given before surgery for analgesia. MI was confirmed at surgery by visual blanching of the left ventricle and changes in the electrocardiogram (ST segment elevation) and validated by terminal echocardiography and infarct sizing by 1% 2,3,5-triphenyltetrazolium chloride (TTC) (Sigma) staining. Mice were randomized to treatment groups and investigators were blinded for data acquisition and analysis.

At the time of MI, recombinant S100A8/A9 (RnD Systems Cat. No. 8916-S8-050; 4.4 ng/g/h or 3.2 µg/day, endotoxin free) was given subcutaneously by osmotic minipump (Alzet Cat. No. 2001 D for *D1* and Cat. No. 1003 D for *D3*). Osmotic minipumps were activated for 3 h for *D1* pumps and overnight for *D3* pumps per manufacturer recommendation. S100A9 was given as a complex with S100A8 because in vivo the proteins exist as a stable heterodimer. The dose used was similar to previous reports (26–28). Saline was infused as the vehicle control. Echocardiography was performed before MI and at MI *D1* and *D3* using the Vevo 3100 (FUJIFILM Visualsonics), in accordance to the guidelines for measuring cardiac physiology in mice (29). Following echocardiography, the mice were euthanized and the tissue was collected, snap frozen, and stored at −80°C. Infarct sizes were calculated as the percentage infarcted area of total LV area using Adobe Photoshop. The LV infarct region was separated from the remote noninfarcted region and stored individually. Sample sizes were *D1* saline (*n* = 6 M), *D1* S100A8/A9 (*n* = 7 M), *D3* saline (*n* = 6 M), and *D3* S100A8/A9 (*n* = 5 M).

### Degranulation Assay

Bone marrow-derived neutrophils were isolated from C57BL/6J male mice (3- to 6- mo old; *n* = 3) using the autoMACS Pro Separator (Miltenyi Biotec) with anti-Ly6G microbeads (Miltenyi Biotech, Cat. No. 130-120-337), as previously described (5). Neutrophils (1.5 × 10^6^ cells) were resuspended in 1 mL RPMI 1640 (Gibco, Cat. No. 21875034), with 1% penicillin-streptomycin (Thermo Fisher Scientific, Cat. No. 15140-122). The cells were stimulated with S100A8/A9 (RnD Systems, Cat. No. 8916-S8-050; 500 ng/mL) for 15 min at 37°C. Unstimulated neutrophils served as negative controls. At the end of stimulation, samples were centrifuged at 800 *g* for 8 min. The secretome (supernatant) and cell pellet were separated, individually snap frozen, and stored at −80°C until use. MMP-9 release into the secretome was measured by immunoblotting, as an indicator of degranulation status.

### Immunoblot Analysis

Immunoblotting was performed according to the published guidelines (30). For immunoblotting of infarcted LV, total protein (10 µg) was loaded on 4%–12% Criterion XT Bis-Tris precast gels (Bio-Rad) and transferred to nitrocellulose membranes using the Trans-Blot Turbo Transfer Pack (Bio-Rad). Membranes were stained with Pierce Reversible Protein Stain Kit (Thermo Fisher Scientific). Membranes were blocked with Blotting Grade Blocker (5%, Bio-Rad) in phosphate buffer solution (PBS) containing 0.1% Tween 20 and incubated overnight at 4°C with the primary antibody (Cedarlane, anti-mouse neutrophils Cat. No. CL8993AP, 1:1,000; Cedarlane, anti-mouse Mac-3 Cat. No. CL8943AP-3, 1:1,000; Invitrogen, anti-mouse S100A8 Cat. No. PA5-79948, 1:1,000; Invitrogen, anti-mouse S100A9 Cat. No. PA1-46489, 1:1,000), followed by incubation at room temperature for 1 h with the secondary antibody (Vector, anti-rat IgG, Cat. No. PI-9400, dilution 1:5,000, anti-rabbit IgG, Cat. No. PI-1000, dilution 1:5,000). Chemiluminescent images were captured using the iBright FL1000 imaging system (Thermo Fisher Scientific) and quantified using iBright analysis software 4.0.0. The blots were normalized to the total protein and the data are presented as normalized arbitrary units. Antibodies were validated using positive controls, including bone marrow-derived neutrophils for the neutrophil Ly6b blot, LV macrophages for the Mac-3 blot, and recombinant mouse MMP-9 for MMP-9 blot. Spleen was used as a standard positive control for all blots.

For immunoblotting of the neutrophil secretome, the above protocol was followed with the following modifications. Equal volume (10 µL) was loaded for each sample, because the cell number per volume was equal. The primary antibody used was against MMP-9 (Millipore Sigma, Cat. No. AB19016, dilution 1:1,000) and the secondary antibody was anti-rabbit IgG (Vector, Cat. No. PI-1000, dilution 1:5,000). The data are presented as arbitrary units.

### Statistics

Statistical analyses were performed with GraphPad Prism 9, according to the guidelines outlined in Statistical Considerations in Reporting Cardiovascular Research (31). Data were reported as means ±SE. Normality was evaluated using the Shapiro–Wilk test and all data passed normality. For correlation analysis, association between two variables was determined by calculating the Pearson correlation coefficient. Survival curves were analyzed by Mantel-cox (log rank) test. The Students *t* test was used to compare two groups: unpaired *t* test was used for the in vivo data and paired *t* test was used for the in vitro data. A *P* value < 0.05 was considered statistically significant.

## RESULTS

### Neutrophil Proteins Positively Correlated with the Wall Thinning Index

**Figure 2.**
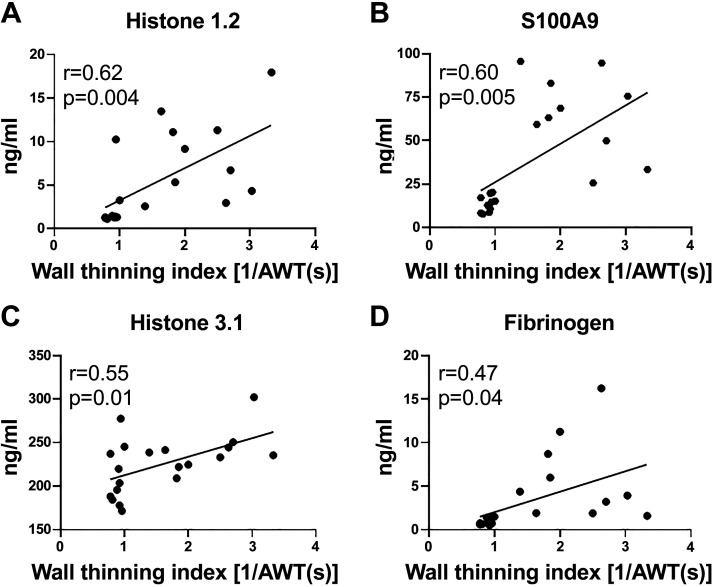
Neutrophil proteins positively correlated with the infarct wall thinning index at MI *day 1*: histone 1.2 (*A*), S100A9 (*B*), histone 3.1 (*C*), and fibrinogen (*D*). MI, myocardial infarction.

Out of 123 proteins examined, four proteins positively correlated with the wall thinning index (Fig. 2). Ranked by *r*, these were histone 1.2, S100A9, histone 3.1, and fibrinogen. The histone proteins identified are intracellular components and likely reflect the change in metabolic status of the neutrophil (32–34). Because S100A9 was the highest ranked secreted factor, we evaluated whether it was causally involved in infarct wall thinning and could serve as a functional effector biomarker.

### S100A8/A9 Administration Increased Neutrophil Infiltration at MI *D1*

**Figure 3.**
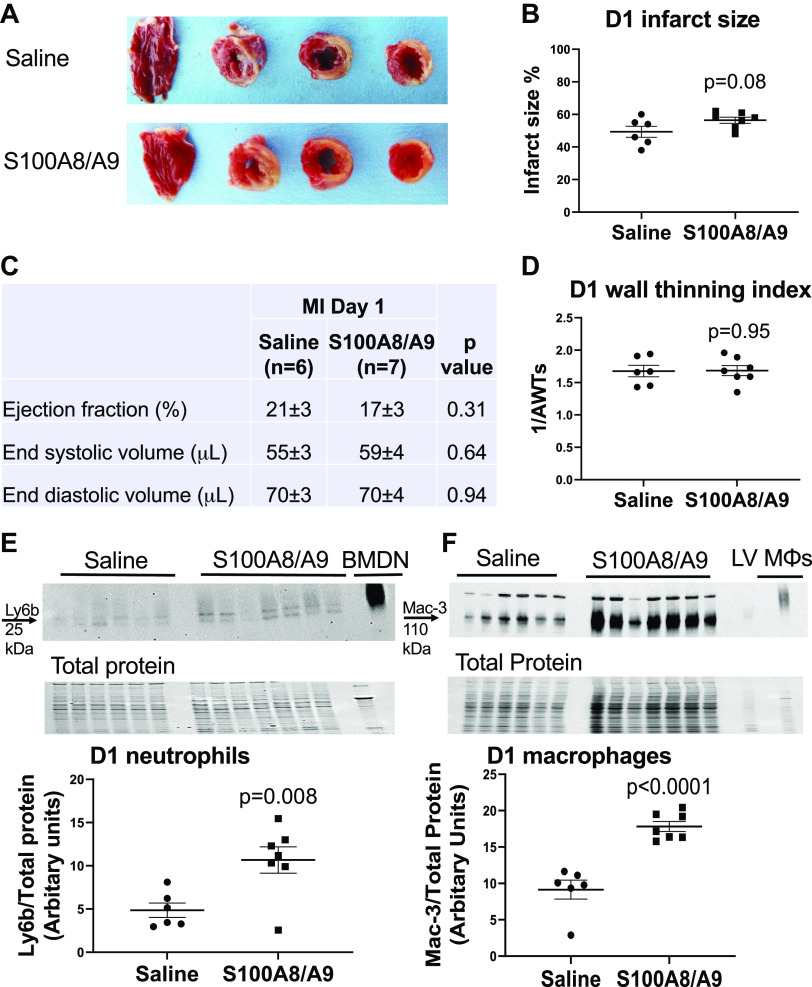
S100A8/A9 robustly increased neutrophil and macrophage infiltration without changing cardiac physiology at MI *day 1*. *A*: representative infarct size images taken after triphenyltetrazolium chloride (TTC) staining of each treatment group. *B*: infarct size was similar between saline and S100A8/A9 groups. *C*: cardiac physiology variables were similar between the saline and S100A8/A9 groups. *D*: infarct wall thinning index (1/AWTs) was similar between the saline and S100A8/A9 groups. *E*: neutrophil (Ly6b expression) infiltration increased in the left ventricle (LV) infarct in the S100A8/A9-infused group compared with saline vehicle control. *F*: macrophage (Mac-3 expression) infiltration increased in the LV infarct in the S100A8/A9-infused group compared with saline vehicle control. Sample sizes were saline (*n* = 6 M) and S100A8/A9 (*n* = 7 M); data analyzed by unpaired *t* test. MI, myocardial infarction.

To test whether exogenous administration of S100A8/A9 stimulated infarct wall thinning, we infused S100A8/A9 beginning at the time of MI. At MI *D1*, neutrophil and macrophage infiltration were measured by immunoblotting and infarct wall thickness by echocardiography. Survival at MI *D1* was identical between MI groups, with 100% of mice enrolled with successful MI surviving to *D1*. Infarct size was also similar in both groups at MI *D1* (Fig. 3, *A* and *B*); the saline group had an infarct size of 49 ± 3% and the S100A8/A9 group 56 ± 3% (*P* = 0.082). Cardiac physiology, including wall thinning index, was also similar between saline and S100A8/A9-treated groups at MI *D1* (Fig. 3, *C* and *D*). To test whether exogenous administration of S100A8/A9 increased neutrophil and macrophages influx to the infarct, we measured ly6b and mac-3 in the LV infarct by immunoblotting. Neutrophil and macrophage infiltration were robustly increased by S100A8/A9 treatment compared with saline control (Fig. 3, *E* and *F*). As expected, there was little to no neutrophil infiltration in the LV remote region and S100A8/A9 had no effect (0.54 ± 0.14 normalized arbitrary units for saline and 0.62 ± 0.13 normalized arbitrary units for S100A8/A9, *P* = 0.66). This indicated that the primary effect of S100A8/A9 at MI *D1* was to amplify proinflammatory neutrophil and macrophage entry to the infarct.

### S100A9 Administration Increased Infarct Wall Thinning at MI *D3*

**Figure 4.**
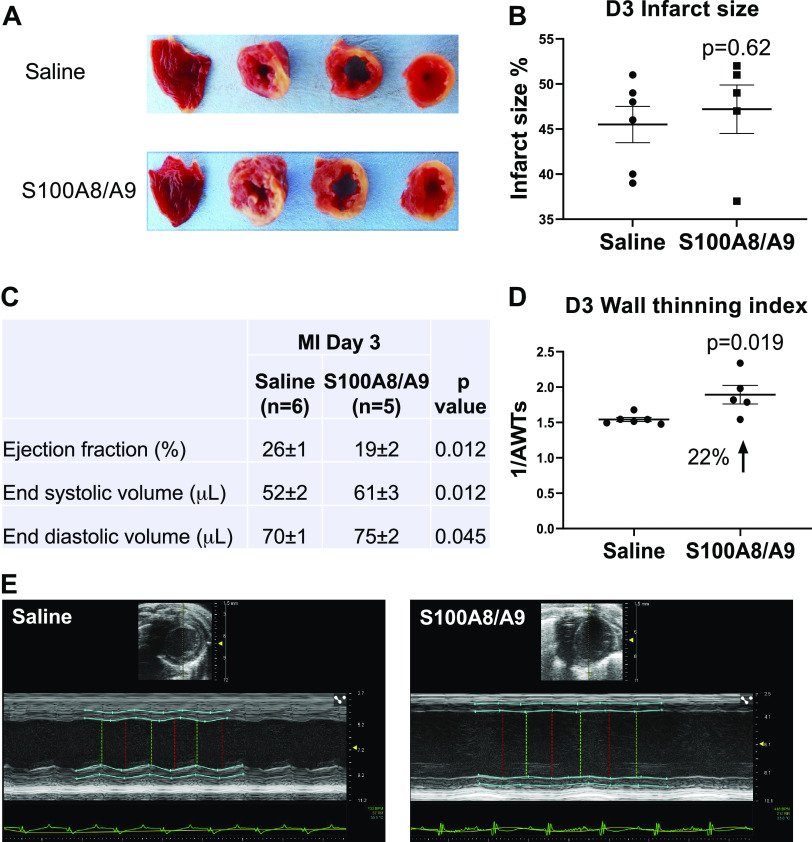
S100A8/A9 increased wall thinning and impaired cardiac physiology after MI without affecting infarct sizes. *A*: representative infarct size images taken after triphenyltetrazolium chloride (TTC) staining of each treatment group. *B*: infarct size was similar between saline and S100A8/A9 groups. *C*: S100A8/A9 exacerbated overall cardiac dysfunction after MI compared with saline treatment. *D*: S100A8/A9 increased the infarct wall thinning index by 22% compared with saline treatment. *E*: representative images of cardiac physiology measurements by two-dimensional (2-D echocardiography; saline (*left*) and S100A8/A9 (*right*). Sample sizes were saline (*n* = 6) and S100A8/A9 (*n* = 7); data analyzed by unpaired *t* test. MI, myocardial infarction.

**Figure 5.**
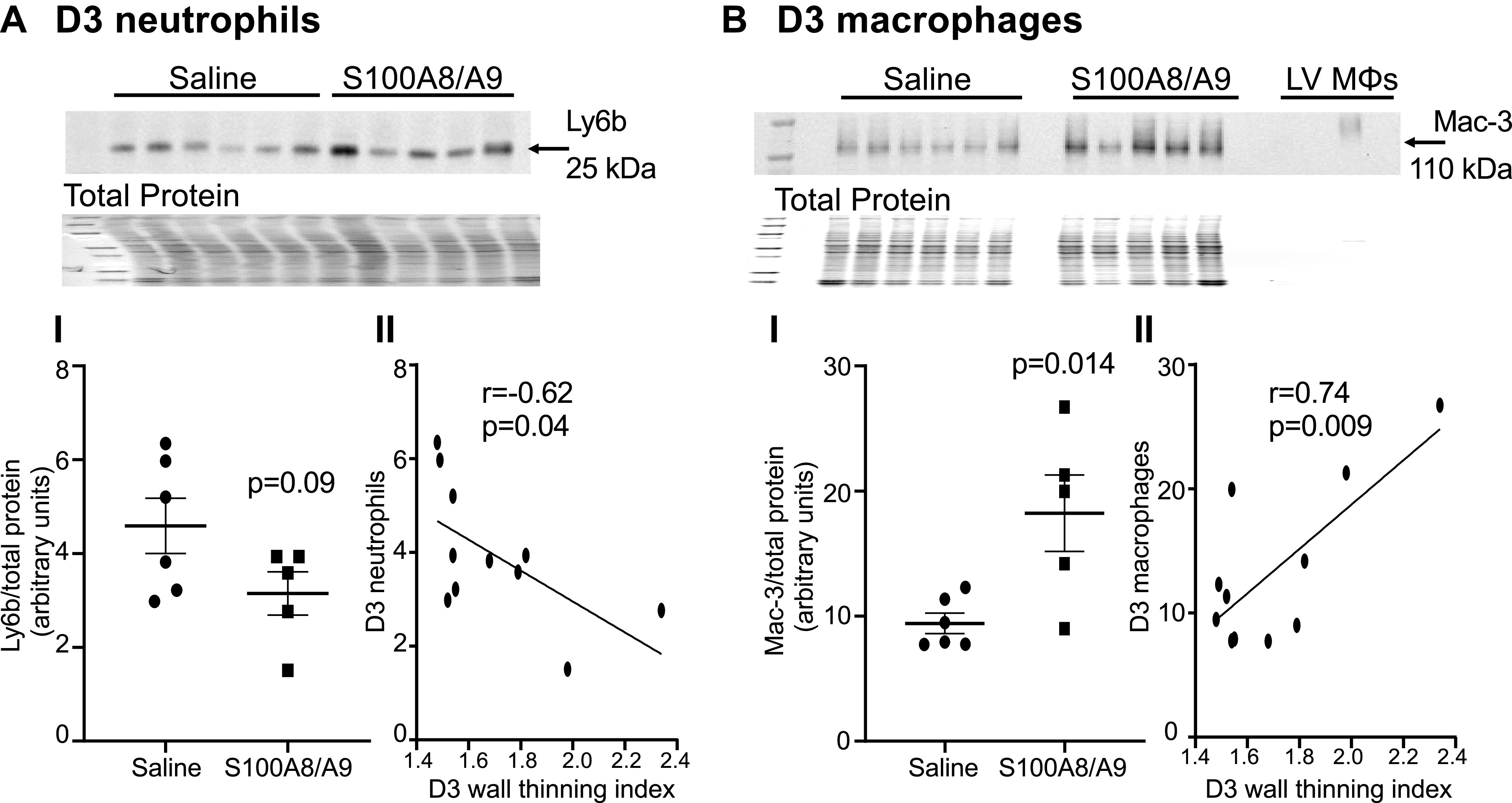
Infarct wall thinning observed at myocardial infarction (MI) *day 3* negatively correlated with neutrophils and positively correlated with macrophages. *A*, *I*: S100A8/A9 did not change neutrophil infiltration (Ly6b expression) in the left ventricle (LV) infarct in the S100A8/A9 group (*n* = 6) compared with saline group (*n* = 5), *P* = 0.09. Data analyzed using unpaired *t* test. *A*, *II*: neutrophils negatively correlated with the infarct wall thinning index at MI *D3*. *B*, *I*: S100A8/A9 increased macrophage infiltration (Mac-3 expression) in the LV infarct in the S100A8/A9 group (*n* = 6) compared with saline group (*n* = 5), *P* = 0.014. Data analyzed using unpaired *t* test. *B*, *II*: macrophages positively correlated with the infarct wall thinning index at MI *D3*. MI, myocardial infarction.

To determine whether the increased neutrophils at MI *D1* would lead to increased infarct wall thinning later, we evaluated MI *D3*. Survival was not different between the saline group (6/6 survived, 100%) and the S100A8/A9 group (5/6 survived, 83%, *P* = 0.31) and infarct sizes were similar (Fig. 4, *A* and *B*). When compared with saline control, S100A8/A9 administration worsened cardiac physiology, as indicated by decreased ejection fraction and increased end systolic and end diastolic volumes (Fig. 4*C*). S100A8/A9 treatment resulted in a 22% increase in the wall thinning index (Fig. 4*D*). By *D3*, neutrophil infiltration was not different between saline and S100A8/A9 groups (Fig. 5*AI*). There continued to be a sustained twofold increase in macrophages in the S100A8/A9 group compared with saline (Fig. 5*BI*). Neutrophil count at MI *D3* negatively correlated with infarct wall thinning index (*r* = −0.62, *P* = 0.04; Fig. 5*AII*), whereas macrophage count at MI *D3* positively correlated with infarct wall thinning index (*r* = 0.74, *P* = 0.009; Fig. 5*BII*). These results indicate that sustained S100A8/A9 treatment increased macrophages at MI *D3* to exacerbate infarct wall thinning.

### S100A8/A9 Did Not Increase MMP-9 in Vivo and Did Not Directly Stimulate Neutrophil Degranulation In Vitro

**Figure 6.**
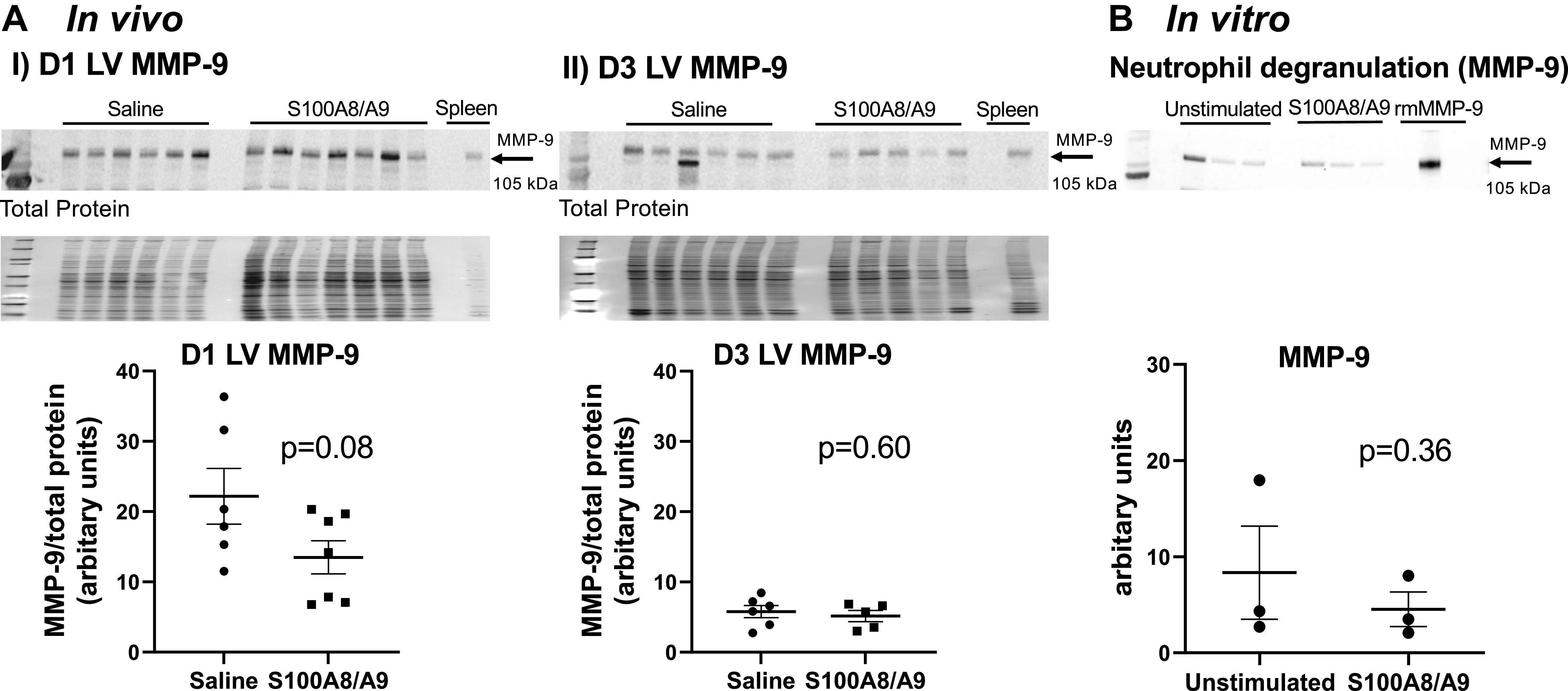
S100A8/A9 did not directly stimulate MMP-9 expression in vivo or neutrophil degranulation in vitro. *A*, *I*: S100A8/A9 did not increase MMP-9 expression in the left ventricle (LV) infarct at myocardial infarction (MI) *D1*. *A*, *II*: S100A8/A9 did not increase MMP-9 expression in the LV infarct at MI *D3*. Sample sizes were saline (*n* = 6) and S100A8/A9 (*n* = 5–7); data analyzed by unpaired *t* test. *B*: S100A8/A9 did not increase MMP-9 release from bone marrow-derived neutrophils stimulated with S100A8/A9 for 15 min in vitro. Samples were *n* = 3; data analyzed using paired *t* test.

To evaluate whether the correlation of S100A9 to infarct wall thinning was linked to MMP-9, we evaluated MMP-9 protein expression in the LV infarct. There were no differences between groups at MI *D1* or *D3* (Fig. 6*A*, *I* and *II*). To determine whether S100A8/A9 had a direct effect on neutrophil degranulation, we stimulated bone marrow-derived neutrophils in vitro with S100A8/A9 and measured MMP-9 release into the secretome as an index of degranulation. As shown in Fig. 6*B*, S100A9 did not directly induce release of MMP-9. This indicates that the exacerbated LV physiology seen due to administration of S100A8/A9 was primarily due to stimulating the influx of proinflammatory leukocytes at MI *D1* and *D3* and through stimulating tissue degrading action of macrophages more than neutrophils.

### S100A8/A9 Activity Increased with Treatment

**Figure 7.**
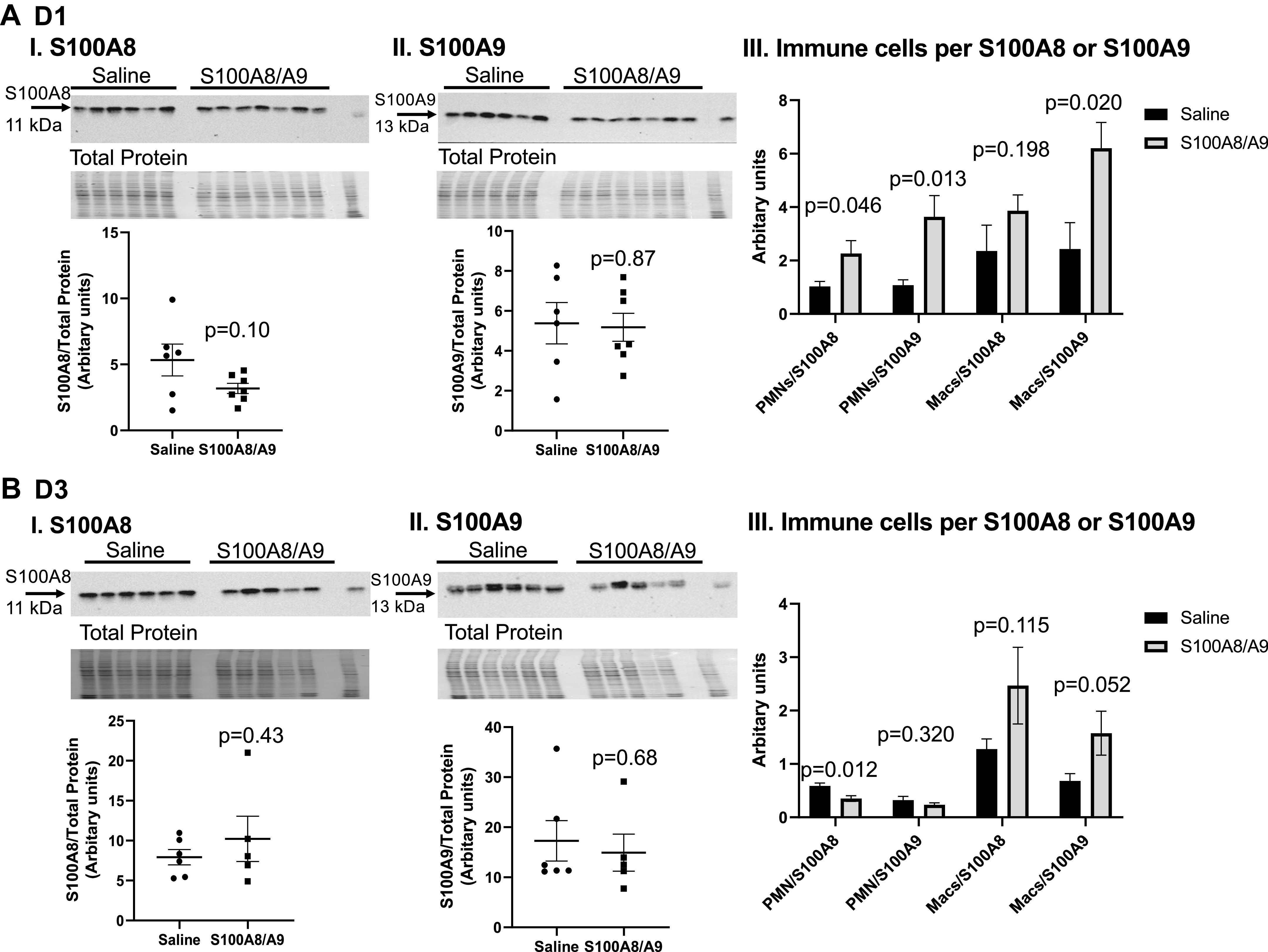
S100A8/A9 administration increased immune cells per S100A8 or S100A9 at myocardial infarction (MI) *D1*. S100A8 (*A*, *I*) or S100A9 (*A*, *II*) expression in the left ventricle (LV) infarct was not elevated at MI *D1*. *A*, *III*: neutrophil-to-S100A8 or -S100A9 and macrophage-to-S100A9 ratios were increased in the S100A8/A9-treated group compared with saline. Samples sizes were saline (*n* = 6) and S100A8/A9 (*n* = 7); data analyzed using unpaired *t* test. S100A8 (*B*, *I*) or S100A9 (*B*, *II*) expression in the LV infarct was not elevated at MI *D3*. *B*, *III*: neutrophil-to-S100A8 ratio decreased at MI *D3*. Samples were saline (*n* = 6) and S100A8/A9 (*n* = 5); data analyzed using unpaired *t* test.

S100A8 or S100A9 was not elevated at MI *D1* or *D3* in the infarct region of the S100A8/A9-treated mice (Fig. 7*, AI* and *II,* and *BI* and *II*). This is consistent with past reports that S100A8/A9 has an autoinhibitory feedback mechanism that turns off its production (35, 36). To measure activity, we calculated the ratio of neutrophils and macrophages (immune cells) per unit of S100A8 or S100A9 expression in the LV infarct (Fig. 7, *AIII* and *BIII*). The neutrophil-to-S100A8 or -S100A9 ratio and the macrophage-to-S100A9 ratio were all higher at MI *D1*, whereas the neutrophil-to-S100A8 ratio was lower at MI *D3*. This indicates that S100A8/A9 activity was higher in treated group compared with control and administration of S100A8/A9 elevates inflammatory cell infiltration early, as its primary mechanism of action for exacerbating LV physiology later in MI.

## DISCUSSION

**Figure 8.**
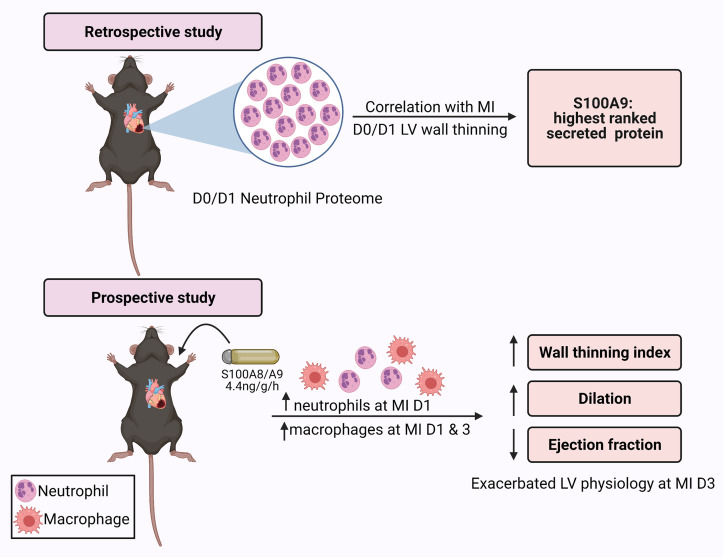
By retrospective evaluation followed by a prospective validation study, S100A9 was determined to be a functional effector of infarct wall thinning. Created with BioRender.com and published with permission.

The goal of this study was to identify proteins derived from the neutrophil that best correlate with infarct wall thinning and serve as causal mediators. We selected to evaluate the relationship between wall thinning and the neutrophil proteome at MI *D1* because both peak at this time point. There were four proteins identified that positively tracked with wall thinning: S100A9, histone 1.2, histone 3.1, and fibrinogen. Of these, S100A9 was the highest ranked secreted factor. By in vivo exogenous S100A8/A9 administration, S100A8/A9 increased leukocyte influx at MI *days 1* and *3* and infarct wall thinning at MI *day 3*. Our results indicate that S100A9 is a functional effector of infarct wall thinning (Fig. 8).

Activated neutrophils secrete DAMPs to maintain inflammatory status by renewing leukocyte infiltration. S100A8/A9 is a proinflammatory alarmin abundantly expressed in neutrophils (6, 37). S100A8/A9 acts through Toll-like receptor 4 (TLR4) and receptor of advanced end glycation end products (RAGE) to activate innate immune responses (14, 35, 38). S100A8/A9 primes the nucleotide-binding oligomerization domain (NOD)-like receptor family pyrin domain-containing protein 3 (NLRP3) inflammasome to produce interleukin (IL)-1β, which leads to myeloid cell recruitment to the infarct (35, 39). Genetic disruption of S100A8/A9 or its downstream signaling mediators improves cardiac physiology after MI (35). A similar study by Marinkovic and colleagues showed that short-term pharmacological inhibition of S100A8/A9 also improves cardiac function at MI *D3*, which is similar to our findings (40).

A primary role of the neutrophil in the infarct is to secrete proteases to remove necrotic myocytes and damaged ECM, which prepares the infarct for scar deposition. Although neutrophils are essential for cardiac wound healing, excessive neutrophils amplify inflammation and promote excessive tissue proteolysis. Neutrophils secrete proteases by degranulation, including MMP-9, which regulates early clearance of the necrotic debris. Our laboratory has shown that neutrophils polarize over the course of MI, with the neutrophil proteome showing a functional shift from proinflammatory to reparative phenotype over the first week in mice (8, 23). This study validates that increased influx of proinflammatory neutrophils, macrophages, and associated proteins after MI *D1* leads to sustained inflammation, which can debilitate the wound healing process. Targeting neutrophil polarization in cancer has shown positive results by decreasing tumor size and may be a potential avenue for MI therapy as well (41). S100A9 could be a neutrophil-specific therapeutic switch point (42).

In the present study, S100A8/A9 increased neutrophil and macrophage infiltration in the infarct at MI *D1*, leading to sustained increase of macrophages and increased wall thinning at MI *D3*. The LV samples were snap frozen and collected for biochemical analysis; as such, we were not able to perform immunohistochemistry to show localization of leukocytes within the infarct region. The effect on infiltration is limited to the infarct region, as the remote region showed little or no influx or neutrophils either with saline or S100A8/A9 treatment, which is consistent with past reports (43).

Our results highlight that the effect of S100A8/A9 is not a direct effect on neutrophil degranulation. Degranulation was defined as MMP-9 release, which is one surrogate marker. Other markers of degranulation include MMP-8, myeloperoxidase, and serine elastase (14). Rather, the effect was indirect in stimulating a greater influx of proinflammatory leukocytes. In addition, S100A9 is a calcium-binding protein and as such could also have a direct effect on the cardiomyocyte sarcomere (44). This would be consistent with the decrease in ejection fraction observed at MI *D3* in the treated group. Our in vivo and in vitro results combined indicate that S100A8/A9 does not itself specifically target wall thinning through neutrophil degranulation.

S100A8/A9 peaks within 6 h of MI and is autoregulated for its activity by the formation of oligotetramers, which competitively bind TLR-4 to block signaling and negatively feedback to reduce further production (35, 36). S100A8/A9 acts as a major inducer for granulopoiesis and mobilization of granulocytes within the infarct (35, 45). We observed that S100A8/A9 induced early changes in immune cell infiltration. S100A8/A9 potentiated inflammation by increasing proinflammatory leukocyte influx into the heart and exacerbated LV dysfunction after MI. S100A8/A9 stimulation of TLR4 activates nuclear factor-κB (NF-κB) to induce translation of various proinflammatory cytokines [e.g., tumor necrosis factor alpha (TNFα), IL-1β, IL-6, and IL-8 (14, 18, 28, 46)]. Serum S100A9 is a potential prognostic marker of major cardiovascular events in patients with acute MI and CXCR2^+^ neutrophils in the heart are the main producers of S100A9 early during reperfusion (47, 48). There is strong evidence, therefore, that S100A9 is an effector marker.

The infarct wall thinning index directly correlated with increased neutrophils and macrophages at MI *D1* and with only macrophages at MI *D3*. Although neutrophil influx was elevated at MI *D1*, macrophage numbers remained elevated at *D3*. Similar to neutrophils, proinflammatory macrophages peak at MI *D1* (22, 23). S100A9 induces a proinflammatory macrophage phenotype in osteoarthritis (49). Macrophages also express S100A9, which is lost during conversion from pro- to anti-inflammatory phenotype (46). Exogenous administration of S100A9 stimulates the transition from inflammatory Ly6C^hi^ monocytes to reparatory Ly6C^lo^ macrophages through upregulation of the transcription factor Nur77 (40, 50).

Increased proinflammatory macrophage influx has been associated with LV rupture (10). MI *D3* macrophage numbers directly correlated with the infarct wall thinning index, which could be either a reflection or consequence of S100A8/A9 treatment or both. MI *D3* neutrophils show a negative correlation with infarct wall thinning, indicating that wall thinning seen with S100A8/A9 administration is due initially to action on the neutrophil and macrophage and later to the potentiation of macrophage activity. S100A9 could be influencing neutrophil to macrophage cross talk, as it is known that neutrophils coordinate the influx of macrophages (51, 52). Disrupted leukocytes kinetics after MI induced by S100A8/A9 could explain the physiological effects observed (53, 54). One limitation of this study was the use of only male mice. Future studies exploring the role of S100A8/A9 on MI remodeling in female mice is warranted.

For this study, we focused on the highest ranked secreted protein, which was S100A9. For the S100A8/A9 (calprotectin) heterodimer complex, only S100A9 was measured in the protein array. The other proteins identified, histone 1.2, histone 3.1, and fibrinogen, were also strong indicators of wall thinning and should be evaluated in future studies. Histones are markers of NETosis and likely reflect intracellular reorganization of the neutrophil at MI *D1* (38). Increased histones suggest formation of extracellular traps and sustenance of inflammation, which is also in line with our findings (55). In a clinical study, Liu et al. (56) showed that neutrophils obtained from infarct-related arteries released NETs and that dsDNA levels were significantly higher in coronary plasma samples and independently associated with in-hospital major adverse cardiac events. Inhibition of peptidyl arginine deiminase (PAD)-4, an enzyme responsible for citrullination of arginine in histones, by PAD4-specific chemical inhibitor GSK484 protected against left ventricular dysfunction after MI (57). Furthermore, excessive NETs is associated with reactive oxygen species (ROS)-dependent aggravation of myocardial injury in MI in apolipoprotein E-deficient mice (58). Nagareddy et al. (38) linked the potential of NETosis to S100A8/A9 release from neutrophils in a recent article, demonstrating a requirement of NETosis in S100A8/A9-induced granulopoiesis.

Fibrinogen is a novel neutrophil marker of degranulation previously identified by our team, which remains upregulated in neutrophil proteome throughout *D7* (8). Increased production of fibrinogen at *D1* of MI by neutrophils indicates a significant role of neutrophils in MI wound healing with key inputs in ECM reorganization. Early secretion of ECM proteins such as fibrinogen from neutrophils could aid in formation of a provisional matrix scaffold for infiltrating neutrophils and monocytes in the infarct, hence sustaining inflammation (59). Fibrinogen is also known to activate fibroblasts and increased fibrinogen early in MI can potentially be linked to fibrosis (60). Fibrinogen has previously been suggested by many as a marker for stroke and MI (61–64). However, this is the first time that fibrinogen has been linked with LV wall thinning and neutrophils and is worth pursuing in future evaluations.

### Conclusions

In summary, S100A9 is an early functional effector of LV infarct wall thinning in the mouse MI model of permanent occlusion. The mechanism of action of S100A9 is directed more at stimulating influx of neutrophils and macrophages rather than direct activation of neutrophil degranulation. Neutrophil proteins are not only potential early biomarkers but also functional effectors for worsened LV physiology after MI and could be therapeutically targeted to promote infarct healing.

## GRANTS

This research was funded by National Institutes of Health Grants GM115458, HL137319, and HL156315; Biomedical Laboratory Research and Development Service of the Veterans Affairs Office of Research and Development Grant 5I01BX000505; American Cancer Society Research Scholar Grant RSG-19–127-01-CSM; the Child Health Research Institute scholars grant at University of Nebraska Medical Center; and Swedish Society for Medical Research Grant P19-0144.

## DISCLAIMERS

The content is solely the responsibility of the authors and does not necessarily represent the official views of any of the funding agencies. All authors have reviewed and approved the article.

## DISCLOSURES

No conflicts of interest, financial or otherwise, are declared by the authors.

## AUTHOR CONTRIBUTIONS

U.C. conceived and designed research; U.C., S.R.K., J.R.R.-P., and D.F. performed experiments; U.C., M.B.-A., S.R.K., J.R.R.-P., and D.F. analyzed data; U.C., M.B.-A., J.D.S., D.R.A., and L.M.C. interpreted results of experiments; U.C. prepared figures; U.C. and M.L.L. drafted manuscript; U.C., M.B.-A., S.R.K., J.R.R.-P., D.F., J.D.S., D.R.A., L.M.C., and M.L.L., edited and revised manuscript; U.C., M.B.-A., M.J.D., S.R.K., J.R.R.-P., D.F., J.D.S., D.R.A., L.M.C., and M.L.L. approved final version of manuscript.
